# Supplementary material for: COZOID: contact zone identifier for visual analysis of protein-protein interactions
Source: BMC Bioinformatics. 2018 Apr 6;19:125. doi: 10.1186/s12859-018-2113-6 (PMC5889581; doi:10.1186/s12859-018-2113-6)
Supplement: Supplementary file 2 — Software build. Executable binary file of the software tool. (ZIP 118,784 kb) [file 12859_2018_2113_MOESM2_ESM.zip › cozoid/java/docs/beansbinding-1.2.1-doc.zip!/index-files/index-23.html]

W-Index (Beans Binding)


---


|  |  |  |
| --- | --- | --- |
| **Overview** \| Package \| Class \| Use \| **Tree** \| **Deprecated** \| **Index** \| **Help** | |  |
| **PREV LETTER**   NEXT LETTER | **FRAMES**    **NO FRAMES**     **All Classes** |  |


A B C D E F G H I J K L M N O P Q R S T U V W 

---


## **W**

**writeExternal(ObjectOutput)** - Method in class org.jdesktop.el.impl.lang.FunctionMapperImpl.Function: **writeExternal(ObjectOutput)** - Method in class org.jdesktop.el.impl.lang.FunctionMapperImpl: **writeExternal(ObjectOutput)** - Method in class org.jdesktop.el.impl.lang.VariableMapperImpl: **writeExternal(ObjectOutput)** - Method in class org.jdesktop.el.impl.MethodExpressionImpl: **writeExternal(ObjectOutput)** - Method in class org.jdesktop.el.impl.MethodExpressionLiteral: **writeExternal(ObjectOutput)** - Method in class org.jdesktop.el.impl.ValueExpressionImpl: **writeExternal(ObjectOutput)** - Method in class org.jdesktop.el.impl.ValueExpressionLiteral

---


|  |  |  |
| --- | --- | --- |
| **Overview** \| Package \| Class \| Use \| **Tree** \| **Deprecated** \| **Index** \| **Help** | |  |
| **PREV LETTER**   NEXT LETTER | **FRAMES**    **NO FRAMES**     **All Classes** |  |


A B C D E F G H I J K L M N O P Q R S T U V W 

---
